# Post-marketing safety surveillance of vortioxetine hydrobromide: a pharmacovigilance study leveraging FAERS database

**DOI:** 10.3389/fpsyt.2025.1532803

**Published:** 2025-02-24

**Authors:** Ying Zhang, Shengzhu Sun, Yunhong Ning

**Affiliations:** First College of Clinical Medicine, Shandong University of Traditional Chinese Medicine, Jinan, Shandong, China

**Keywords:** vortioxetine hydrobromide, adverse events, data mining, major depressive disorders, FAERS

## Abstract

**Background:**

Vortioxetine hydrobromide is a widely prescribed medication for the treatment of major depressive disorder (MDD), primarily exerting its antidepressant effects by inhibiting the reuptake of serotonin (5-HT).The objective of this study was to investigate adverse events (AEs) associated with vortioxetine hydrobromide through data mining in the FDA Adverse Event Reporting System (FAERS) to enhance clinical safety.

**Methods:**

We collected FAERS data from Q3 2013 to Q1 2024 for data cleansing. Disproportionality analysis was employed to quantify relevant AEs associated with vortioxetine. Reported Ratio of Ratios (ROR) was utilized for identifying risk signals within the FAERS data. We employed the System Organ Classes (SOCs) and selected the Preferred Terms (PTs) from the Medical Dictionary for Regulatory Activities (MedDRA version 26.1).

**Results:**

A total of 11,298 cases were reported as "primary suspected (PS)" for vortioxetine hydrobromide. Notably, at the systemic organ level (SOC) level, the adverse effects associated with vortioxetine hydrobromide involved 27 systemic organoid classes (SOCs).We identified 150 significantly disproportionate Preferred Terms (PTs) that met all four algorithms.

**Conclusion:**

This study identified adverse events (AEs) associated with vortioxetine. Our findings offer valuable insights for optimizing the use of vortioxetine hydrobromide and reducing potential side effects, serving as a reference for its rational and safe clinical application.

## 1 Introduction

Major depressive disorder (MDD) is regarded as a significant global health concern due to its severity, affecting approximately 300 million people worldwide (1). The disorder is characterized by at least one depressive episode lasting up to two weeks, including depressed mood, lack of interest or anhedonia, difficulty concentrating, cognitive challenges, fatigue, sleep disturbances, and suicidal thoughts (2, 3). In the general population, the lifetime prevalence of MDD is 4.4%, which places a significant burden on individuals, society, and the health care system (4). Although the pathophysiology of MDD remains incompletely understood, current studies suggest that this complex is multifactorial, involving neurotransmitter dysregulation, inflammatory responses, genetic factors, environmental influences, and neuroplastic changes (2, 5–7). Moreover, oxidative stress is closely related to the pathophysiology of MDD (8) and plays a significant role in neurodegeneration (9), contributing to the pathogenesis of MDD.

In September 2013, vortioxetine hydrobromide (referred to as vortioxetine in the following text ) was approved by the FDA for the treatment of MDD (10). Vortioxetine is a multimodal antidepressant characterized by several unique mechanisms of action (11).In addition to its use for treating MDD, vortioxetine is effective in alleviating depressive symptoms in patients with epilepsy (12), addressing bipolar depression (13), and managing depressive symptoms in elderly patients (14). Vortioxetine is well-tolerated in clinical settings and is rarely associated with serious adverse reactions (15). It interacts with various serotonin receptors, exhibiting antagonistic effects on 5-HT_3_, 5-HT_1D_, and 5-HT_7_ receptors. These properties may help regulate neurotransmitter activity in the central nervous system, affect serotonin transmission between neurons, and play a role in mood and cognitive function regulation. Additionally, vortioxetine partially stimulates 5-HT_1B_ receptors, which may be linked to improvements in mood, anxiety, and cognitive function, while also acting as an agonist on 5-HT_1A_ receptors. Compared to selective serotonin reuptake inhibitors (SSRIs), vortioxetine has a distinct mechanism of action that produces antidepressant effects by directly modulating serotonin receptor activity and inhibiting serotonin transporters (16–20). Anna Julia Krupa et al. pointed out that vortioxetine is not a typical 5-HT_3_ antagonist, and it may produce an excitatory effect in the early stage of treatment, and then switch to antagonistic effect after reaching a steady state, which can explain the common nausea or vomiting in the early. The antagonistic effect on 5-HT_7_ synergistically with the agonistic effect on 5-HT_1A_, enhancing the desensitization of 5-HT_1A_ autoreceptors and increasing serotoninergic transmission. Additionally, the antagonistic effect on 5-HT_1B_ receptors is associated with enhanced antidepressant and anxiolytic effects, potentially offsetting the risk of weight gain. Furthermore, they suggest that increasing the dosage in clinical practice could improve the drug's effectiveness (15).

This study used the latest reported data from the FDA's Adverse Event Reporting System (FAERS) database to evaluate AEs associated with the use of vortioxetine in FAERS from Q3 2013 to Q1 2024 for pharmacovigilance analysis. The ICSR (Individual Case Safety Reports) database offers a wealth of individual case information, enabling us to conduct a thorough analysis of the adverse reactions associated with vortioxetine. By systematically collecting and collating case reports from different sources, serves as a crucial foundation for establishing connections between drug safety and specific adverse effects. Furthermore, by employing disproportionality analysis, we can identify potential patterns in the occurrence of these adverse effects and assess the risk profile of vortioxetine. This approach effectively addresses the knowledge gap regarding the adverse effects of vortioxetine. The findings from this study can assist clinicians in monitoring adverse drug reactions and offer recommendations for the safe clinical use of vortioxetine.

## 2 Data sources and methods

### 2.1 Data sources and procedures

**Figure 1:**
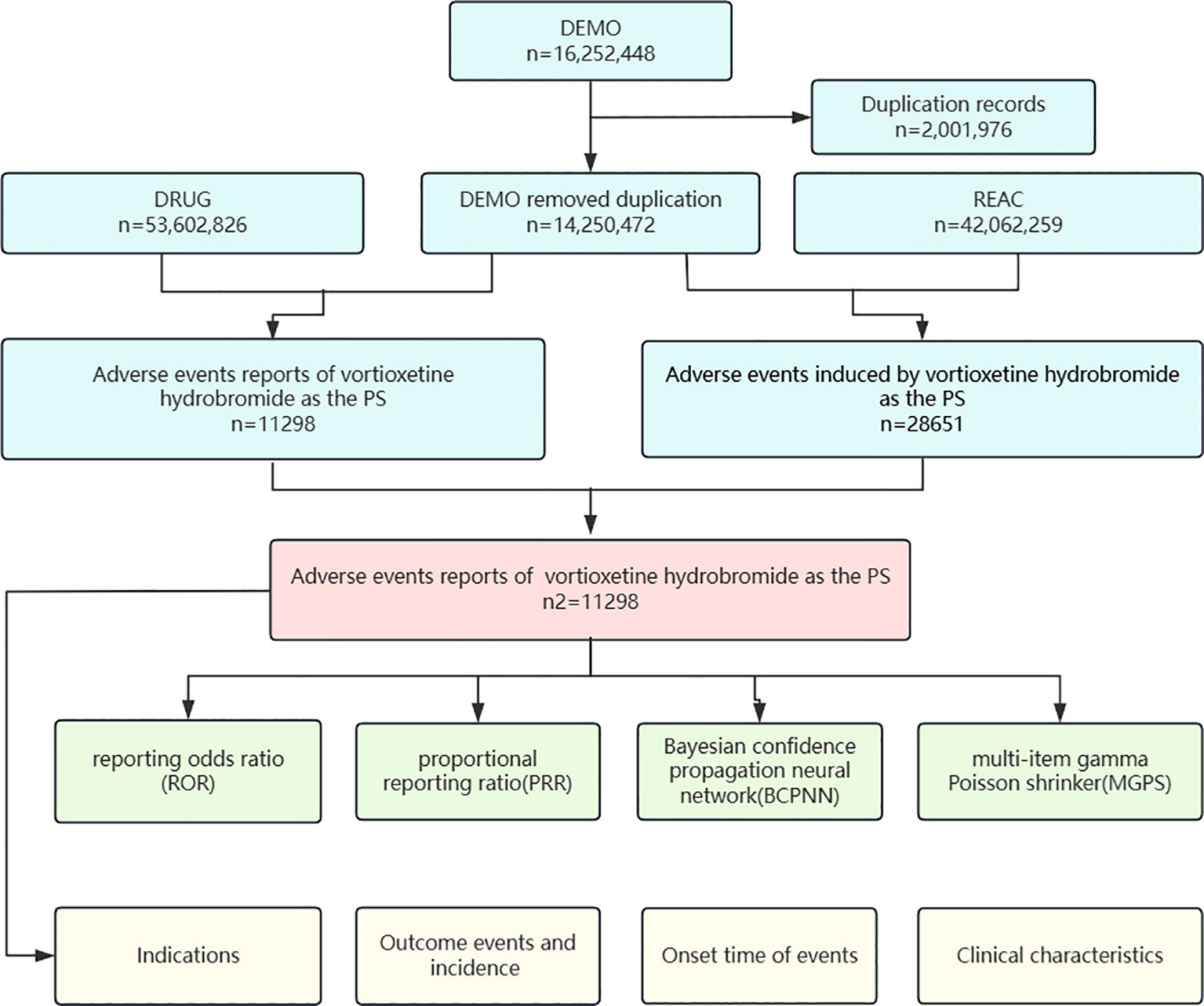
Proceed of selecting cases of Vortioxetine Hydrobromide Aes from the FAERS detabase. n= Vortioxetine Hydrobromide. DEMO, demographic and administrative information; DRUG, drug information; EAC, adverse drug reaction information; PS, the primary suspect drug.

The FAERS database is a publicly available database of self-reported adverse reactions from physicians, pharmacists, lawyers, consumers, and drug manufacturers from many countries around the world, with quarterly updated data. The vortioxetine study data were derived from the FAERS Adverse Drug Reaction Reporting Database from Q3 2013 to Q1 2024. We used generic names (trintellix) and ingredient names (vortioxetine) to identify ADEs associated with vortioxetine hydrobromide. We extracted a total of 16,252,448 reports for pharmacovigilance analysis. The overall data were derived from the DEMO, Drug Use Record Form (DRUG), Patient Adverse Event Record Form (REAC) and Patient Outcome Information Form (OUTC). In accordance with the FDA's recommended method for deduplication, we selected the relevant fields: the main number (PRIMARYID), case number (CASEID), and date and time (FDA_DT) from the Patient Personal Information Form (DEMO). We sorted these records by CASEID, FDA_DT, and PRIMARYID, retaining reports with the most recent FDA_DT value when CASEIDs were the same. For reports where both CASEID and FDA_DT were identical, we kept the entry with the highest PRIMARYID value. Ultimately, we included 11,298 reports for further analysis (**Figure 1**).

### 2.2 Statistical analysis

The system organ class (SOC) and preferred terms (PT) in the medical dictionary of regulatory activities (MedDRA) version 26.1 adverse drug reaction terminology set were used to classify and describe the adverse event (AE) reports of vortioxetine. We used Reporting Odds Ratio (ROR), Proportional Reporting Ratio (PRR), Multi item Gamma Poisson Shrinker (MGPS), and Bayesian Confidence Propagation Neural Network (BCPNN) to explore the adverse reaction (ADR) signals of vortioxetine.

### 2.3 Data Mining

The proportion imbalance method was used as the main analysis method to determine whether it is a positive signal according to the reporting odds ratio (ROR) algorithm. The signal generation rule is that the target drug-related risk signal inclusion criteria is the number of cases N≥3, lower limit of 95% CI>1; according to the proportional reporting ratio (PRR) algorithm, judge whether it is a positive signal, and the signal generation rule is PRR≥2,x^2^≥4, N≥3; according to the multi item gamma Poisson shrinker (MGPS) algorithm, judge whether it is a positive signal, and the signal generation rule is EBGM05>2. According to the Bayesian confidence propagation neural network (BCPNN) algorithm, determine whether it is a positive signal, and the signal generation rule is IC025>0. The equations and criteria of these four algorithms are shown in **Table 1**. If the signal is generated, it indicates that there is a statistical correlation between the target drug and AE. There is a correlation between the two. The larger the value, the stronger the signal, the greater the correlation between the target drug and AE. The four algorithms are combined to reduce the result bias caused by a single algorithm.

**Table 1:** Four major algorithms used for signal detection.

Algorithms	Equation	Criteria
ROR	ROR=ad/b/c	lower limit of 95% CI>1, N≥3
	95%CI=e^ln(ROR)±1.96(1/a+1/b+1/c+1/d)^0.5^	
PRR	PRR=a(c+d)/c/(a+b)	PRR≥2, χ^2^≥4, N≥3
	χ^2^=[(ad-bc)^2](a+b+c+d)/[(a+b)(c+d)(a+c)(b+d)]	
BCPNN	IC=log_2_a(a+b+c+d)(a+c)(a+b)	IC025>0
	95%CI= E(IC) ± 2V(IC)^0.5	
MGPS	EBGM=a(a+b+c+d)/(a+c)/(a+b)	EBGM05>2
	95%CI=e^ln(EBGM)±1.96(1/a+1/b+1/c+1/d)^0.5^	

Equation: a, number of reports containing both the target drug and target adverse drug reaction; b, number of reports containing other adverse drug reaction of the target drug; c, number of reports containing the target adverse drug reaction of other drugs; d, number of reports containing other drugs and other adverse drug reactions. 95%CI, 95% confidence interval; N, the number of reports; χ^2^, chi-squared; IC, information component; IC025, the lower limit of 95% CI of the IC; E(IC), the IC expectations; V(IC), the variance of IC; EBGM, empirical Bayesian geometric mean; EBGM05, the lower limit of 95% CI of EBGM.

## 3 Results

### 3.1 Characteristics of adverse event reports

We extracted all reported cases from the FAERS database from Q3 2013 to Q1 2024, totaling 16,252,448 cases. After data cleaning and screening, we obtained 11,298 reports of adverse reactions related to vortioxetine. The reported data were analyzed in this study, and the general characteristics of the AEs associated are listed in **Table 2**.

**Table 2:** Summary of basic demographic and clinical information of reports associated with vortioxetine hydrobromide based on the FAERS database (From 1 July 2013 to 31 March 2024).[cases(%)].

Characteristics	Case number, n	Case proportion, %
**Number of events**	**11298**	
Gender		
Female	6875	60.9%
Male	2937	26.0%
Missing	1486	13.2%
Age		
<18	123	1.1%
18~64.9	3932	34.8%
65~85	850	7.5%
>85	67	0.6%
Missing	6326	56.0%
Weight		
<50 kg	137	1.2%
>100 kg	331	2.9%
50~100 kg	1868	16.5%
Missing	8962	79.3%
Reported person (Top 5)		
Consumer	5679	50.3%
Physician	2966	26.3%
Health Professional	752	6.7%
Pharmacist	282	2.5%
Other health-professional	1318	11.7%
Reported countries (Top 3)		
United States	9010	79.7%
Japan	491	4.3%
France	310	2.7%
Outcome		
Hospitalization - Initial or Prolonged	961	8.5%
Death	236	2.1%
Disability	132	1.2%
Life-Threatening	154	1.4%
Congenital Anomaly	11	0.1%
Permanent Impairment	6	0.1%
Other Serious (Important Medical Event)	2035	18.0%
missing	7763	68.7%

The number of reports identifying the gender of the submitter was 9,812, with the number of reports from women (n=6,875, 60.9%) higher than that from men (n=2,937, 26.0%). We found age-specific information in 4,972 reports, which included 123 cases (1.1%) involving individuals under 18 years old, 3,932 (34.8%) aged 18-64, 850 (7.5%) aged 65-84, and 67 (0.6%) over 85 years old. Of the weight data involving 2,336 patients, the largest proportion (16.5%) was in the 50-100 kg group.

**Figure 2:**
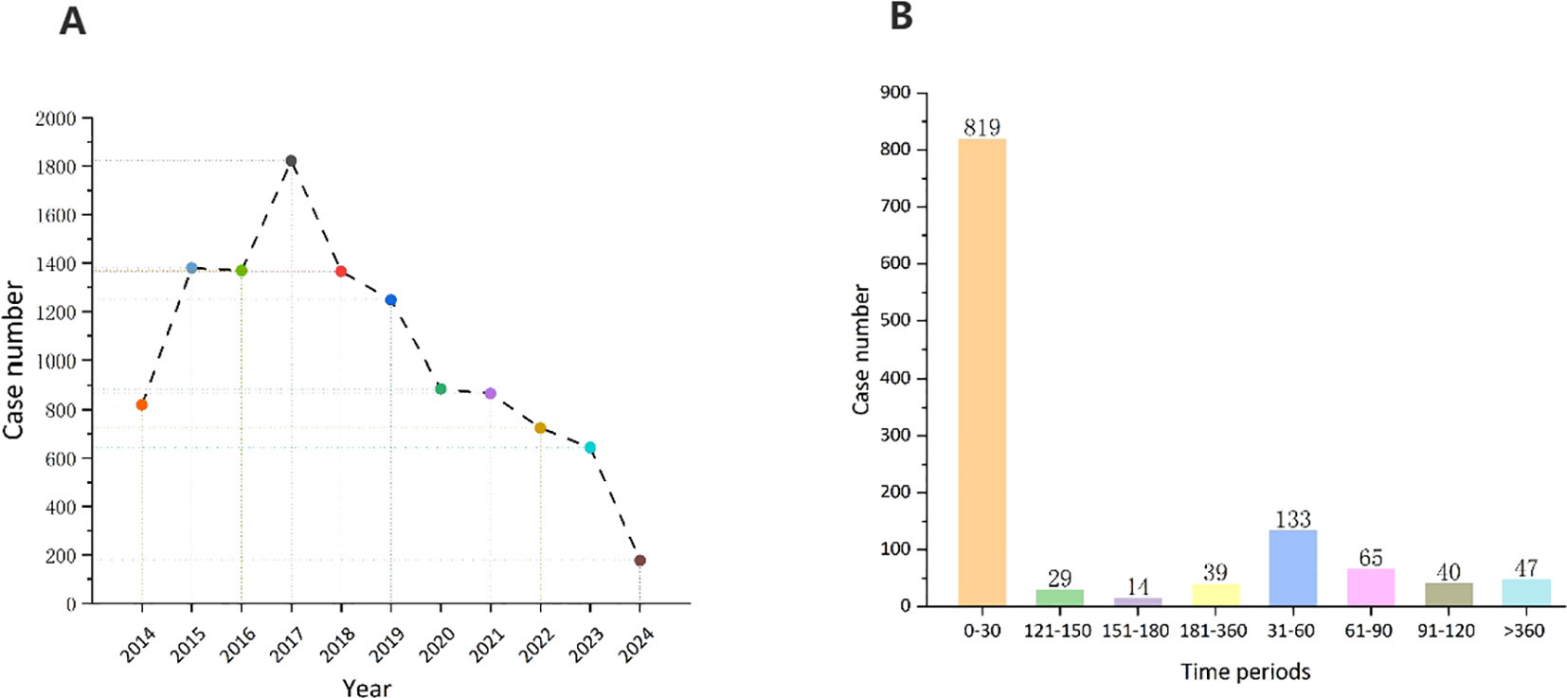
**(A)** Distribution of ADEs of vortioxetine from Q3 2013 to Q1 2024. **(B)** Time to onset (TTO) analysis (counted in days) of vortioxetine related ADEs at the overall level.

Most adverse reaction reports were submitted by consumers (n=5,679, 50.3%), and the country with the highest number of reports was the United States (n=9,010, 79.7%). **Figure 2A** shows the annual distribution of vortioxetine-related adverse reactions. The highest number of reports was reported in 2017 (1,822 reports), after which the number of adverse reaction reports has steadily declined.

### 3.2 Onset time of events

After excluding inaccurate, missing, or unknown reports of onset, we collected the time to onset of adverse events related to vortioxetine from the database. The median time to onset was 7 days [IQR, 4-41 days]. As shown in **Figure 2B**, the majority of cases (60.0%) occurred within the first month of vortioxetine administration. Over time, the number of reported adverse reactions has decreased. It is important to note that even after 1 year of treatment with vortioxetine, adverse drug events may still occur.

### 3.3 Signal of system organ class

**Figure 3:**
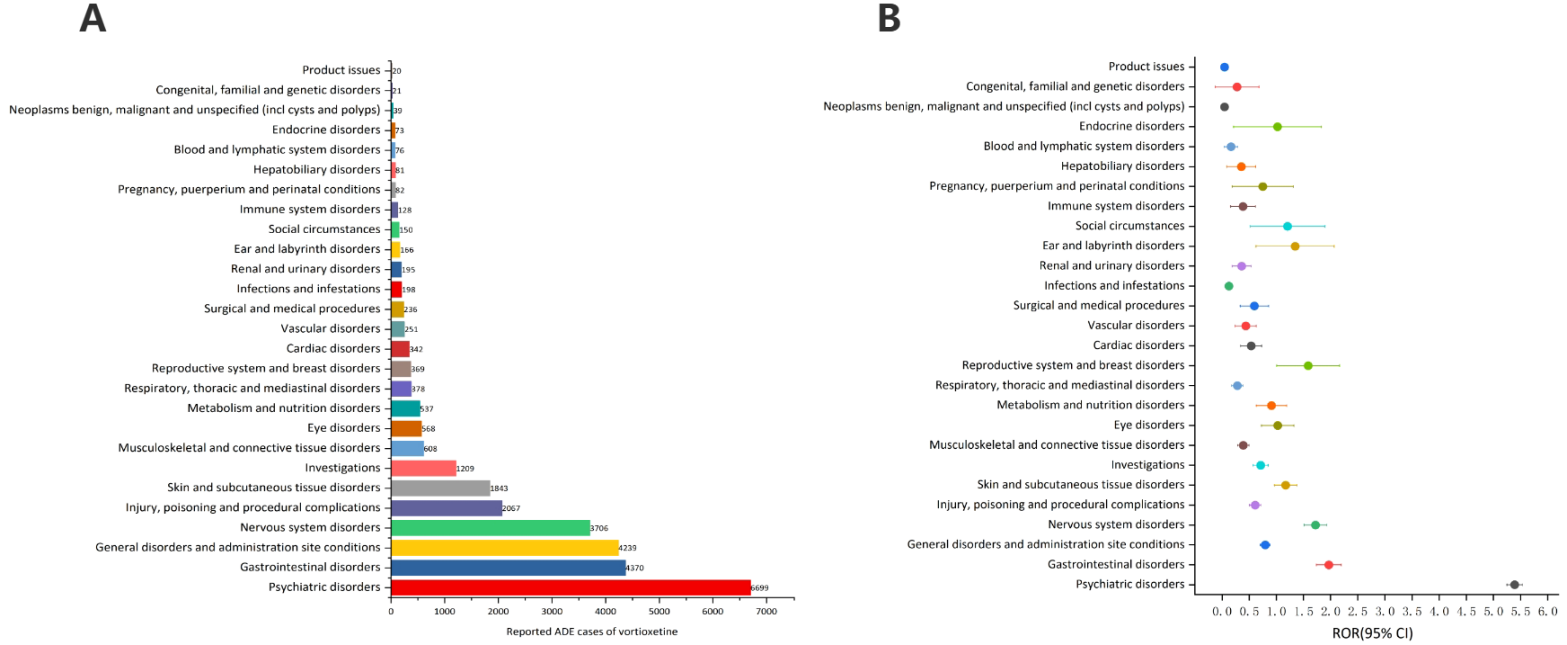
Signals detection at the SOC level. **(A)** The bar chart displays the reported cases of ADEs at each SOC level. **(B)** The ROR values and their 95% confidence intervals (95% CI) are visualized. ADEs, adverse drug events; SOC, System Organ Class; ROR, reporting odds ratio.

**Table 3** describes the signal intensity and reporting of vortioxetine at the SOC level. Vortioxetine-related adverse events were reported across 27 organ systems. The number of case reports of vortioxetine-associated SOCs is illustrated in **Figure 3A**. The highest number of reports in SOC occurred in psychiatric disorders (SOC: 10037175, n=6,699). Significant SOCs that meet at least one criterion among the four indicators include psychiatric disorders (SOC:10037175, n=6,699), gastrointestinal disorders (SOC:10017947, n=4,370), nervous system disorders (SOC:10029205, n=3,706), skin and subcutaneous tissue disorders (SOC:10040785, n=1,843), reproductive system and breast disorders (SOC:10038604, n=369), ear and labyrinth disorders (SOC:10013993, n=166), social circumstances (SOC:10041244, n=150. Notably, psychiatric disorders are SOCs that meet all four criteria simultaneously (**Table 3**). **Figure 3B** shows the ROR of the vortioxetine-associated SOC signal strength with its 95% confidence interval.

**Table 3:** Signal strength of reports of vortioxetine hydrobromide at the System Organ Class (SOC) level in FAERS database.

System organ class (SOC)	Vortioxetine hydrobromide cases reporting SOC	ROR (95% two-side Cl)	PRR (χ2)	EBGM(EBGM05)	IC(IC025)
Psychiatric disorders	6699	5.39 ( 5.24 - 5.54 )*	4.36 ( 18298.24 )*	4.35 ( 4.25 )*	2.12 ( 0.46 )*
Gastrointestinal disorders	4370	1.97 ( 1.9 - 2.03 )*	1.82 ( 1758.16 )	1.82 ( 1.77 )	0.86 ( -0.8 )
General disorders and administration site conditions	4239	0.8 ( 0.77 - 0.82 )	0.83 ( 190.06 )	0.83 ( 0.8 )	-0.28 ( -1.94 )
Nervous system disorders	3706	1.72 ( 1.66 - 1.78 )*	1.63 ( 970.45 )	1.63 ( 1.58 )	0.7 ( -0.96 )
Injury, poisoning and procedural complications	2067	0.61 ( 0.58 - 0.64 )	0.64 ( 480.61 )	0.64 ( 0.61 )	-0.65 ( -2.32 )
Skin and subcutaneous tissue disorders	1843	1.17 ( 1.11 - 1.23 )*	1.16 ( 41.95 )	1.16 ( 1.11 )	0.21 ( -1.45 )
Investigations	1209	0.71 ( 0.67 - 0.75 )	0.72 ( 135.64 )	0.72 ( 0.69 )	-0.47 ( -2.13 )
Musculoskeletal and connective tissue disorders	608	0.39 ( 0.36 - 0.42 )	0.4 ( 568.34 )	0.4 ( 0.38 )	-1.31 ( -2.98 )
Eye disorders	568	1.02 ( 0.94 - 1.11 )	1.02 ( 0.25 )	1.02 ( 0.95 )	0.03 ( -1.64 )
Metabolism and nutrition disorders	537	0.91 ( 0.83 - 0.99 )	0.91 ( 5.14 )	0.91 ( 0.85 )	-0.14 ( -1.81 )
Respiratory, thoracic and mediastinal disorders	378	0.28 ( 0.25 - 0.31 )	0.29 ( 707.61 )	0.29 ( 0.26 )	-1.81 ( -3.47 )
Reproductive system and breast disorders	369	1.58 ( 1.42 - 1.75 )*	1.57 ( 76.73 )	1.57 ( 1.44 )	0.65 ( -1.02 )
Cardiac disorders	342	0.53 ( 0.48 - 0.59 )	0.53 ( 141.69 )	0.53 ( 0.49 )	-0.9 ( -2.57 )
Vascular disorders	251	0.43 ( 0.38 - 0.49 )	0.44 ( 184.22 )	0.44 ( 0.4 )	-1.19 ( -2.86 )
Surgical and medical procedures	236	0.59 ( 0.52 - 0.67 )	0.6 ( 65.75 )	0.6 ( 0.54 )	-0.75 ( -2.41 )
Infections and infestations	198	0.12 ( 0.11 - 0.14 )	0.13 ( 1246.24 )	0.13 ( 0.11 )	-2.97 ( -4.63 )
Renal and urinary disorders	195	0.35 ( 0.31 - 0.41 )	0.36 ( 230.21 )	0.36 ( 0.32 )	-1.49 ( -3.15 )
Ear and labyrinth disorders	166	1.33 ( 1.14 - 1.55 )*	1.32 ( 13.26 )	1.32 ( 1.17 )	0.41 ( -1.26 )
Social circumstances	150	1.19 ( 1.01 - 1.4 )*	1.19 ( 4.46 )	1.19 ( 1.04 )	0.25 ( -1.42 )
Immune system disorders	128	0.38 ( 0.32 - 0.45 )	0.38 ( 130.02 )	0.38 ( 0.33 )	-1.39 ( -3.06 )
Pregnancy, puerperium and perinatal conditions	82	0.73 ( 0.59 - 0.91 )	0.73 ( 7.92 )	0.73 ( 0.61 )	-0.45 ( -2.11 )
Hepatobiliary disorders	81	0.34 ( 0.28 - 0.43 )	0.35 ( 101.04 )	0.35 ( 0.29 )	-1.53 ( -3.2 )
Blood and lymphatic system disorders	76	0.16 ( 0.13 - 0.2 )	0.16 ( 332.95 )	0.16 ( 0.13 )	-2.62 ( -4.29 )
Endocrine disorders	73	0.99 ( 0.79 - 1.25 )	0.99 ( 0.01 )	0.99 ( 0.82 )	-0.01 ( -1.68 )
Neoplasms benign, malignant and unspecified (incl cysts and polyps)	39	0.05 ( 0.03 - 0.06 )	0.05 ( 767.45 )	0.05 ( 0.04 )	-4.4 ( -6.06 )
Congenital, familial and genetic disorders	21	0.25 ( 0.16 - 0.39 )	0.25 ( 46.62 )	0.25 ( 0.18 )	-1.99 ( -3.65 )
Product issues	20	0.04 ( 0.03 - 0.06 )	0.04 ( 464.35 )	0.04 ( 0.03 )	-4.63 ( -6.3 )

*Indicates statistically signifcant signals in algorithm. ROR, reporting odds ratio; CI confdence interval; PRR proportional reporting ratio; χ2, chi-squared; IC, information component; IC025, the lower limit of 95% CI, of the IC; EBGM, empirical Bayesian geometric mean; EBGM05, the lower limit of 95% CI of EBGM.

### 3.4 Signal of preferred terms

**Figure 4:**
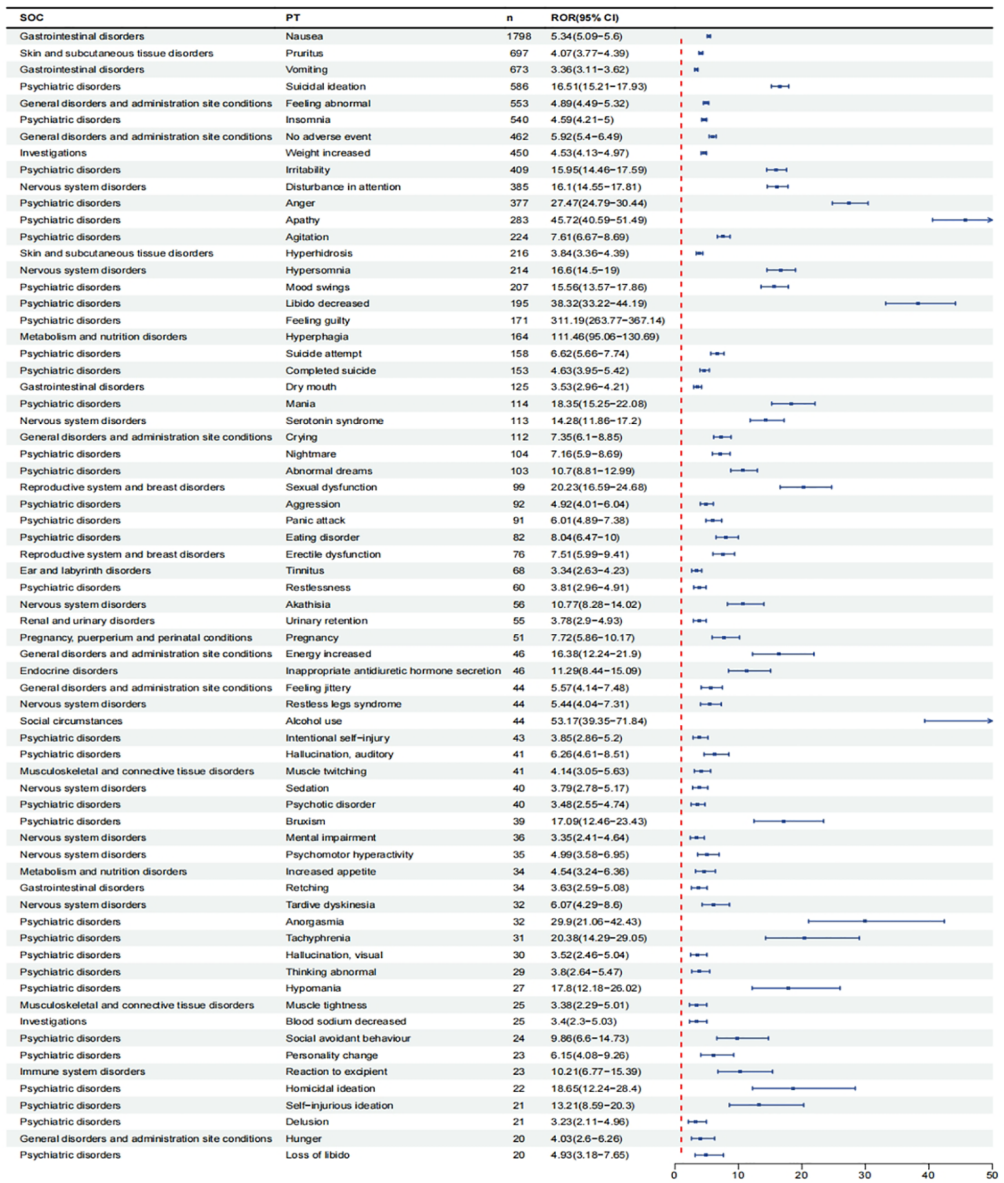
Signals detection at the PT level. We selected PTs with a minimum of 20 cases and displayed the ROR and corresponding 95%Cl using a forest plot.

After excluding PT as a possible indication for vortioxetine, we found a total of 150 significantly disproportionate PTs satisfying all four algorithms simultaneously, as shown in **Supplementary Table 1**. Next, we categorized PTs with more than 20 ADE cases and selected 68 ADEs that met this screening criterion. To improve visualization, we present PT signals in the format of a forest plot and arrange them in descending order by the number of cases (**Figure 4**).These data were then grouped by SOC, with overall results presented in **Table 4**. We identified that PT entries with more than 100 cases included nausea (PT:10028813, n=1,798), vomiting (PT:10047700, n=673), dry mouth (PT:10013781, n=125), suicidal ideation (PT:10042458, n=586), irritability (PT:10022998, n=409), anger (PT:10002368, n=377), agitation (PT:10001497, n=224), mood swings (PT:10027951, n=207), libido decreased (PT:10024419, n=195), suicide attempt (PT:10042464, n=158), completed suicide (PT:10010144, n=153), mania (PT:10026749, n=114), hyperhidrosis (PT:10020642, n=216), weight increased (PT:10047899, n=450), disturbance in attention (PT:10013496, n=385), serotonin syndrome (PT: 10040108, n=113), which are consistent with drug warnings in the drug labels. Interestingly, unexpected significant ADEs were identified, and PTs with more than 50 reports included insomnia (PT:10022437, n=540), apathy(PT:10002942, n=283), feeling guilty(PT:10049708, n=171),nightmare (PT:10029412, n=104), abnormal dreams ( PT:10000125, n=103), pruritus (PT:10037087, n=697), feeling abnormal (PT:10016322, n=553), no adverse event (PT:10067482, n=462) , crying (PT:10011469, n=112), hypersomnia (PT:10020765, n=214), hyperphagia (PT:10020710, n=164), panic attack (PT:10033664, n=91), tinnitus (PT:10043882, n=68), urinary retention (PT:10046555, n=55).

**Table 4:** Signal strength of reports of vortioxetine hydrobromide at the preferred term (PT) level in FAERS database.

System organ class (SOC)	Preferred terms (PTs)	Vortioxetine cases reporting PT	ROR (95% two-sided Cl)	PRR (χ2)	EBGM(EBGM05)	IC(IC025)
Gastrointestinal disorders	Nausea	1798	5.34 ( 5.09 - 5.6 )	5.06 ( 5918.7 )	5.05 ( 4.85 )	2.34 ( 0.67 )
	Vomiting	673	3.36 ( 3.11 - 3.62 )	3.3 ( 1085.67 )	3.3 ( 3.09 )	1.72 ( 0.06 )
	Dry mouth	125	3.53 ( 2.96 - 4.21 )	3.52 ( 225.41 )	3.52 ( 3.03 )	1.81 ( 0.15 )
	Retching*	34	3.63 ( 2.59 - 5.08 )	3.62 ( 64.44 )	3.62 ( 2.73 )	1.85 ( 0.19 )
Psychiatric disorders	Suicidal ideation	586	16.51 ( 15.21 - 17.93 )	16.2 ( 8274.18 )	16.03 ( 14.96 )	4 ( 2.34 )
	Insomnia*	540	4.59 ( 4.21 - 5 )	4.52 ( 1482.26 )	4.51 ( 4.2 )	2.17 ( 0.51 )
	Irritability	409	15.95 ( 14.46 - 17.59 )	15.74 ( 5589.52 )	15.58 ( 14.35 )	3.96 ( 2.3 )
	Anger	377	27.47 ( 24.79 - 30.44 )	27.12 ( 9317.23 )	26.65 ( 24.46 )	4.74 ( 3.07 )
	Apathy*	283	45.72 ( 40.59 - 51.49 )	45.28 ( 11889.36 )	43.95 ( 39.79 )	5.46 ( 3.79 )
	Agitation	224	7.61 ( 6.67 - 8.69 )	7.56 ( 1270.17 )	7.53 ( 6.74 )	2.91 ( 1.25 )
	Mood swings	207	15.56 ( 13.57 - 17.86 )	15.46 ( 2771.64 )	15.31 ( 13.65 )	3.94 ( 2.27 )
	Libido decreased	195	38.32 ( 33.22 - 44.19 )	38.06 ( 6860.73 )	37.13 ( 32.95 )	5.21 ( 3.55 )
	Feeling guilty*	171	311.19 ( 263.77 - 367.14 )	309.34 ( 43404.55 )	255.65 ( 222.62 )	8 ( 6.33 )
	Suicide attempt	158	6.62 ( 5.66 - 7.74 )	6.59 ( 746.26 )	6.56 ( 5.76 )	2.71 ( 1.05 )
	Completed suicide	153	4.63 ( 3.95 - 5.42 )	4.61 ( 431.21 )	4.6 ( 4.02 )	2.2 ( 0.53 )
	Mania	114	18.35 ( 15.25 - 22.08 )	18.28 ( 1839.7 )	18.07 ( 15.48 )	4.18 ( 2.51 )
	Nightmare*	104	7.16 ( 5.9 - 8.69 )	7.14 ( 546.59 )	7.11 ( 6.05 )	2.83 ( 1.16 )
	Abnormal dreams*	103	10.7 ( 8.81 - 12.99 )	10.66 ( 895.93 )	10.6 ( 9.01 )	3.41 ( 1.74 )
	Aggression	92	4.92 ( 4.01 - 6.04 )	4.9 ( 285.16 )	4.89 ( 4.12 )	2.29 ( 0.62 )
	Panic attack*	91	6.01 ( 4.89 - 7.38 )	5.99 ( 377.13 )	5.97 ( 5.03 )	2.58 ( 0.91 )
	Eating disorder	82	8.04 ( 6.47 - 10 )	8.02 ( 501.53 )	7.98 ( 6.66 )	3 ( 1.33 )
	Restlessness	60	3.81 ( 2.96 - 4.91 )	3.81 ( 123.94 )	3.8 ( 3.07 )	1.93 ( 0.26 )
	Intentional self-injury	43	3.85 ( 2.86 - 5.2 )	3.85 ( 90.43 )	3.84 ( 2.99 )	1.94 ( 0.28 )
	Hallucination, auditory	41	6.26 ( 4.61 - 8.51 )	6.26 ( 180.29 )	6.23 ( 4.82 )	2.64 ( 0.97 )
	Psychotic disorder	40	3.48 ( 2.55 - 4.74 )	3.47 ( 70.28 )	3.47 ( 2.67 )	1.79 ( 0.13 )
	Bruxism*	39	17.09 ( 12.46 - 23.43 )	17.06 ( 583.06 )	16.88 ( 12.96 )	4.08 ( 2.41 )
	Anorgasmia	32	29.9 ( 21.06 - 42.43 )	29.86 ( 874.96 )	29.29 ( 21.85 )	4.87 ( 3.2 )
	Tachyphrenia*	31	20.38 ( 14.29 - 29.05 )	20.36 ( 562.84 )	20.09 ( 14.93 )	4.33 ( 2.66 )
	Hallucination, visual	30	3.52 ( 2.46 - 5.04 )	3.52 ( 53.9 )	3.51 ( 2.6 )	1.81 ( 0.15 )
	Thinking abnormal*	29	3.8 ( 2.64 - 5.47 )	3.8 ( 59.64 )	3.79 ( 2.79 )	1.92 ( 0.26 )
	Hypomania	27	17.8 ( 12.18 - 26.02 )	17.79 ( 422.66 )	17.59 ( 12.8 )	4.14 ( 2.47 )
	Social avoidant behaviour*	24	9.86 ( 6.6 - 14.73 )	9.85 ( 189.56 )	9.79 ( 7 )	3.29 ( 1.62 )
	Personality change	23	6.15 ( 4.08 - 9.26 )	6.14 ( 98.63 )	6.12 ( 4.34 )	2.61 ( 0.95 )
	Homicidal ideation*	22	18.65 ( 12.24 - 28.4 )	18.64 ( 362.56 )	18.41 ( 12.95 )	4.2 ( 2.54 )
	Self-injurious ideation	21	13.21 ( 8.59 - 20.3 )	13.2 ( 234.69 )	13.09 ( 9.14 )	3.71 ( 2.04 )
	Delusion*	21	3.23 ( 2.11 - 4.96 )	3.23 ( 32.3 )	3.23 ( 2.25 )	1.69 ( 0.02 )
	Loss of libido	20	4.93 ( 3.18 - 7.65 )	4.93 ( 62.45 )	4.92 ( 3.4 )	2.3 ( 0.63 )
Skin and subcutaneous tissue disorders	Pruritus*	697	4.07 ( 3.77 - 4.39 )	3.99 ( 1569.2 )	3.99 ( 3.74 )	1.99 ( 0.33 )
	Hyperhidrosis	216	3.84 ( 3.36 - 4.39 )	3.82 ( 449.67 )	3.81 ( 3.41 )	1.93 ( 0.27 )
General disorders and administration site conditions	Feeling abnormal*	553	4.89 ( 4.49 - 5.32 )	4.81 ( 1670.47 )	4.8 ( 4.47 )	2.26 ( 0.6 )
	No adverse event*	462	5.92 ( 5.4 - 6.49 )	5.84 ( 1849.4 )	5.82 ( 5.39 )	2.54 ( 0.87 )
	Crying*	112	7.35 ( 6.1 - 8.85 )	7.32 ( 608.98 )	7.29 ( 6.24 )	2.87 ( 1.2 )
	Energy increased*	46	16.38 ( 12.24 - 21.9 )	16.35 ( 655.78 )	16.18 ( 12.69 )	4.02 ( 2.35 )
	Feeling jittery	44	5.57 ( 4.14 - 7.48 )	5.56 ( 163.92 )	5.54 ( 4.32 )	2.47 ( 0.8 )
	Hunger*	20	4.03 ( 2.6 - 6.26 )	4.03 ( 45.45 )	4.02 ( 2.79 )	2.01 ( 0.34 )
Investigations	Weight increased	450	4.53 ( 4.13 - 4.97 )	4.47 ( 1214.8 )	4.46 ( 4.13 )	2.16 ( 0.49 )
	Blood sodium decreased	25	3.4 ( 2.3 - 5.03 )	3.4 ( 42.19 )	3.39 ( 2.44 )	1.76 ( 0.1 )
Nervous system disorders	Disturbance in attention	385	16.1 ( 14.55 - 17.81 )	15.89 ( 5320.58 )	15.73 ( 14.46 )	3.98 ( 2.31 )
	Hypersomnia*	214	16.6 ( 14.5 - 19 )	16.48 ( 3078.88 )	16.31 ( 14.56 )	4.03 ( 2.36 )
	Serotonin syndrome	113	14.28 ( 11.86 - 17.2 )	14.23 ( 1377.25 )	14.11 ( 12.08 )	3.82 ( 2.15 )
	Akathisia	56	10.77 ( 8.28 - 14.02 )	10.75 ( 491.97 )	10.68 ( 8.57 )	3.42 ( 1.75 )
	Restless legs syndrome*	44	5.44 ( 4.04 - 7.31 )	5.43 ( 158.46 )	5.41 ( 4.22 )	2.44 ( 0.77 )
	Sedation*	40	3.79 ( 2.78 - 5.17 )	3.79 ( 81.89 )	3.78 ( 2.92 )	1.92 ( 0.25 )
	Mental impairment*	36	3.35 ( 2.41 - 4.64 )	3.35 ( 59.07 )	3.34 ( 2.54 )	1.74 ( 0.07 )
	Psychomotor hyperactivity*	35	4.99 ( 3.58 - 6.95 )	4.98 ( 111.03 )	4.97 ( 3.76 )	2.31 ( 0.65 )
	Tardive dyskinesia*	32	6.07 ( 4.29 - 8.6 )	6.07 ( 134.91 )	6.05 ( 4.52 )	2.6 ( 0.93 )
Metabolism and nutrition disorders	Hyperphagia*	164	111.46 ( 95.06 - 130.69 )	110.83 ( 16596.21 )	103.11 ( 90.26 )	6.69 ( 5.02 )
	Increased appetite*	34	4.54 ( 3.24 - 6.36 )	4.54 ( 93.54 )	4.53 ( 3.42 )	2.18 ( 0.51 )
Reproductive system and breast disorders	Sexual dysfunction	99	20.23 ( 16.59 - 24.68 )	20.17 ( 1779.29 )	19.91 ( 16.86 )	4.32 ( 2.65 )
	Erectile dysfunction	76	7.51 ( 5.99 - 9.41 )	7.49 ( 425.57 )	7.46 ( 6.18 )	2.9 ( 1.23 )
Ear and labyrinth disorders	Tinnitus*	68	3.34 ( 2.63 - 4.23 )	3.33 ( 110.77 )	3.33 ( 2.72 )	1.73 ( 0.07 )
Renal and urinary disorders	Urinary retention*	55	3.78 ( 2.9 - 4.93 )	3.77 ( 111.9 )	3.77 ( 3.02 )	1.91 ( 0.25 )
Pregnancy, puerperium and perinatal conditions	Pregnancy*	51	7.72 ( 5.86 - 10.17 )	7.71 ( 296.24 )	7.67 ( 6.09 )	2.94 ( 1.27 )
Endocrine disorders	Inappropriate antidiuretic hormone secretion*	46	11.29 ( 8.44 - 15.09 )	11.27 ( 427.38 )	11.19 ( 8.78 )	3.48 ( 1.82 )
Social circumstances	Alcohol use*	44	53.17 ( 39.35 - 71.84 )	53.09 ( 2170.13 )	51.27 ( 39.85 )	5.68 ( 4.01 )
Musculoskeletal and connective tissue disorders	Muscle twitching	41	4.14 ( 3.05 - 5.63 )	4.13 ( 97.2 )	4.13 ( 3.19 )	2.04 ( 0.38 )
	Muscle tightness*	25	3.38 ( 2.29 - 5.01 )	3.38 ( 41.87 )	3.38 ( 2.43 )	1.76 ( 0.09 )
Immune system disorders	Reaction to excipient*	23	10.21 ( 6.77 - 15.39 )	10.2 ( 189.63 )	10.14 ( 7.19 )	3.34 ( 1.68 )

*Emerging fndings of vortioxetine hydrobromide associated AEs from FAERS database. We selected PTs with a minimum of 20 cases. ROR, reporting odds ratio; CI, confdence interval; PRR, proportional reporting ratio; χ, chi-squared; IC, information component; IC025, the lower limit of 95% CI of the IC; EBGM, empirical Bayesian geometric mean; EBGM05, the lower limit of 95% CI of EBGM.

## 4 Discussion

Vortioxetine was approved by the FDA in 2023 for the treatment of patients with MDD. Given the limited real-world evidence regarding adverse events associated with vortioxetine, we conducted a post-market surveillance study to evaluate the adverse events related to the use of vortioxetine in the FAERS database from Q3 2013 to Q1 2024.

In this study, women reported more adverse events than men, which is broadly consistent with the epidemiological findings of MDD (21, 22). Some researchers suggest that women are more significantly affected by MDD and may respond differently to antidepressant treatments (22). This may be linked to specific experiences such as menstruation, pregnancy, and postpartum (23–25). Additionally, clinical studies have shown that the incidence of MDD in women is twice that of men (26), potentially leading to increased use of vortioxetine among women, resulting in a larger proportion of women in adverse event reports. As the clinical use of vortioxetine rises, it is crucial for clinicians to remain vigilant regarding adverse events associated with this medication. Enhanced drug safety monitoring in clinical practice is essential to reduce the occurrence of serious adverse events.

At the SOC level, the most significant signal is psychiatric disorders, which can be obtained by disproportionate analysis. In addition, the gastrointestinal system and nervous system also rank prominently in the statistics of SOC, which is consistent with many clinical studies. Many studies report that post-treatment adverse effects frequently occur in the psychiatric, neurological, and gastrointestinal systems (19, 27). The validity of this study was confirmed and suggested that the results of the study may accurately reflect real-world clinical practice. At the PT level, the most serious adverse events included nausea, vomiting, dry mouth, suicidal ideation, insomnia, irritability, anger, apathy, agitation, mood swings, libido decreased, feeling guilty, suicide attempt, completed suicide,mania,nightmare, abnormal dreams, pruritus, hyperhidrosis, feeling abnormal, crying, weight increased, disturbance in attention, hypersomnia, serotonin syndrome, and hyperphagia. Among these, there are adverse reactions align with the clinical safety data for the drug (28), including nausea, vomiting, dry mouth, suicidal ideation, irritability, anger, agitation, mood swings, libido decreased, suicide attempt, completed suicide, mania, pruritus, weight increased, disturbance in attention, and serotonin syndrome. Additionally, we identified several valuable new adverse events, including insomnia, apathy, feeling guilty, nightmare, abnormal dreams, panic attack, pruritus, feeling abnormal, no adverse events, crying, hypersomnia, hyperphagia, tinnitus, and urinary retention. These incidental events are often difficult to detect in small-scale clinical trials.

Disproportionate reports indicate a high risk to the psychiatric system, with suicidal thoughts being the most frequently reported adverse event. This is also clearly mentioned in the label. It is important to note that depression itself is a risk factor for suicidal thoughts and behaviors (29). Although suicidal behavior is not always clearly evident in clinical practice, this study's adverse event reports revealed a high prevalence of suicidal ideation. Therefore, we recommend that clinicians utilize standardized assessment scales, such as the Hamilton Depression Rating Scale (HAMD) and the Generalized Anxiety Disorder 7-item scale (GAD-7), to comprehensively evaluate a patient’s mental status before initiating treatment with vortioxetine. During the initial treatment phase (the first 4 to 6 weeks), we suggest conducting weekly or bi-weekly follow-up visits to monitor for side effects and assess symptom improvement. Suicidal ideation and behaviors can also be evaluated using a suicide risk assessment scale, such as the Columbia-Suicide Severity Rating Scale (C-SSRS). After this initial period, the frequency of follow-up visits should gradually lengthen, and dosage adjustments can be made based on the patient’s symptoms and side effects.

In addition, adverse events such as insomnia, aggression, restlessness, anger, hostility, and irritability have occurred in mental illness, and the relevant mechanisms have not been clearly determined. We hypothesize that these issues may be related to the regulation of 5-HT reuptake and effects of vortioxetine on 5-HT receptors. The role of serotonin in the prefrontal cortex, amygdala, and other relevant brain regions is crucial for emotional stability and impulse control (30). In some cases, increasing 5-HT levels with Vortioxetine may trigger a physiological stress response, potentially resulting in mood swings and heightened behavioral responses, particularly in individuals with pre-existing aggressive tendencies.

The most common adverse reactions associated with vortioxetine treatment involve the gastrointestinal system, including nausea, vomiting, and dry mouth. These adverse reactions are frequently noted in the vortioxetine label and clinical trials (31). 5-HT is synthesized and distributed mainly in the central nervous system and intestinal tract (32). Adverse events in the gastrointestinal tract are often associated with 5-HT reuptake inhibitors, and evidence has been found in previous literature (33). Within the gastrointestinal tract exists a distinct nervous system known as the enteric nervous system, which comprises numerous neurons capable of independent regulation from the central nervous system (CNS). Vortioxetine affects nerve conduction in the gastrointestinal tract by altering 5-HT levels, resulting in abnormal intestinal motility. It is suggested that when we use vortioxetine in clinical practice, we need to pay more attention to the protection of the patient's gastrointestinal tract. This includes minimizing the use of medications that also stimulate the gastrointestinal tract during the treatment period and considering dose reductions for patients who experience severe adverse reactions.

In addition, we focused on identifying new and significant adverse reactions that are not included in the drug label. In our study, we found adverse events such as apathy, feeling guilty, abnormal dreams, pruritus, and feeling abnormal. There is currently no very clear mechanism for explaining the relationship between 5-HT and these adverse events. However, it is well established that 5-HT can inhibit REM (rapid eye movement) sleep (REMS) (34). According to Seth C Hopkins et al., vortioxetine may cause a significant reduction in REM sleep while increasing REM latency (35). This may be related to vortioxetine's ability to elevate 5-HT levels, which inhibits REM sleep and promotes deep sleep, potentially leading to a rebound effect in REM sleep. Vortioxetine may affect the histamine system in some cases (10), which could contribute to pruritus. We found that no adverse events accounted for a large proportion of studies, suggesting that vortioxetine is safe and well tolerated in clinical practice. Therefore, clinicians may consider prioritizing the use of vortioxetine in their treatment options.

FAERs database is an important tool, which collects AE reports related to drug treatment, helps to monitor and identify potential adverse events of drugs and promote drug safety. The information is open and transparent, which is of great help to discover and solve drug safety problems in time. However, faers has some limitations. Firstly, not all drug-related adverse events are reported to FDA, which leads to potential underreporting, over reporting and information gaps. Secondly, the data collected by FAERs come from various sources, including clinicians, nurses, health care providers, pharmaceutical companies, patients and patients' families, and there is a lack of many important information when reporting, which leads to analysis bias. When reporting adverse events, these sources may have different views, goals and emotions, which may lead to inconsistencies and disputes in the information. This study did not consider the impact of other drugs on adverse reactions during treatment, so this analysis only represents the correlation between drugs and AEs, and does not represent a causal relationship between reported AEs and specific drugs.

## 5 Conclusion

In this study, the FAERs database was used for pharmacovigilance analysis of vortioxetine hydrobromide. By detecting the adverse reactions of SOC and Pt levels, some adverse reactions not mentioned in the instructions were provided. Our results emphasize that clinicians need to understand not only the adverse reactions mentioned in the instructions, but also some adverse reactions beyond the instructions. This study provides a reference for the rational and safe clinical application of vortioxetine hydrobromide.

## Data Availability

The original contributions presented in the study are included in the article/**Supplementary Material**. Further inquiries can be directed to the corresponding author.
